# Isolated Unilateral Proptosis as a Heralding Feature of Ipsilateral Temporal Dural Arteriovenous Fistula

**DOI:** 10.7759/cureus.117001

**Published:** 2026-09-27

**Authors:** Vaibhav Bhandari, Divyani Garg, Ayush Agarwal, Abhishek Anand, Achal Kumar Srivastava

**Affiliations:** 1 Department of Neurology, All India Institute of Medical Sciences, New Delhi, IND

**Keywords:** brain arteriovenous malformations, dural arteriovenous fistula, orbital proptosis, posterior fossa arteriovenous fistula, unilateral proptosis

## Abstract

Dural arteriovenous fistulas (dAVFs) are rare intracranial vascular abnormalities that may present with varied and sometimes atypical clinical features. While proptosis is commonly associated with anterior cranial or cavernous sinus fistulas, it is unusual in posterior fossa dAVFs.

We present the case of a 41-year-old man with progressive redness, swelling, and protrusion of the left eye over six months, without associated pain, diplopia, headache, or cranial nerve deficits. Ocular examination revealed preserved visual acuity and extraocular movements, with normal fundus findings. An initial diagnosis of a cavernous sinus fistula was considered. However, brain MRI with time-of-flight sequences revealed a left temporal dAVF with feeders from the external carotid artery and drainage into the vein of Labbé and transverse sinus, with a possible connection to the superior division of the middle cerebral artery. On clinical re-examination, a bruit was noted in the left occipitotemporal region. Doppler and transcranial Doppler studies confirmed mixed arterial and venous flow. The patient was referred for digital subtraction angiography and embolization.

The symptoms of dAVFs vary depending on the shunt characteristics, location, and venous drainage pattern. Ocular signs are uncommon in noncavernous dAVFs. In this case, venous reflux through the vein of Labbé may explain the isolated proptosis. Management is guided by angioarchitecture and associated risk features, with high-risk fistulas requiring timely intervention. This case highlights the need to consider cranial dural AVFs in the differential diagnosis of isolated proptosis, as timely diagnosis and treatment are crucial.

## Introduction

Rare dural-based pathologic shunts known as dural arteriovenous fistulas (dAVFs) account for 10%-15% of all intracranial vascular malformations [1]. dAVFs are anatomically categorized by location, i.e., anterior cranial fossa, cavernous sinus, tentorium cerebelli, superior sagittal sinus, and transverse-sigmoid sinus dAVFs; but clinical behavior and management are more reliably predicted by the angiographic Cognard [1] and Borden [2] classification systems, which grade lesions by the presence of retrograde sinus flow and cortical venous drainage (CVD) rather than by anatomical site alone. The clinical features of dAVFs are generally determined by their location and venous drainage pattern [3]. Proptosis is a common finding in anterior fossa and cavernous sinus dAVFs, whereas posterior fossa dAVFs more typically present with pulsatile tinnitus [3]. Noncavernous dAVFs presenting with isolated ophthalmic symptoms are considerably rare. We report an unusual case of a temporal dAVF presenting as isolated unilateral proptosis and discuss the presumptive angiographic risk classification of the lesion based on noninvasive imaging alone.

## Case presentation

History

A 41-year-old man presented with progressive complaints of redness, swelling, and protrusion of the left eyeball over six months. He did not report headache, diplopia, or ocular pain. There was no history of systemic complaints, including fever, weight loss, cough, or prior cranial trauma, and no medical comorbidities.

Examination

Visual acuity was 6/6 bilaterally with no blurring of vision. Fundus examination and pupillary light reflex were normal, with no relative afferent pupillary defect and normal accommodation. Trigeminal (V1/V2) sensation was intact bilaterally, with no corneal reflex asymmetry. No ocular bruit was appreciated on this initial ocular examination. Given the absence of cranial nerve involvement, an orbital or retro-orbital mass lesion was considered the leading differential at this stage; this was later excluded on MRI, which showed no orbital mass but instead vascular flow voids. The patient was evaluated for other causes of isolated unilateral proptosis, including thyroid eye disease, orbital inflammatory diseases such as sarcoidosis and Wegener's granulomatosis, orbital tumors, and vascular causes such as carotid-cavernous fistula and cavernous sinus thrombosis. Routine blood investigations, including thyroid profile, were normal.

Investigations

MRI brain demonstrated a left-sided temporal intracranial dAVF. Individual sequences showed the following: T2-weighted coronal imaging: swelling in the left orbit; susceptibility-weighted imaging: asymmetrically increased vascularity on the left compared to the right; time-of-flight magnetic resonance angiography: multiple dilated flow voids at the left posterior temporal lobe and around the left transverse sinus, with cortical dilated venous channels along the left temporal lobe and a dilated vein of Labbé; and T1 post-contrast imaging: abnormal enhancing vascular channels along the left posterior temporal region, with the appearance raising the possibility of communication with the superior division of the left middle cerebral artery (Figure 1). Dedicated orbital imaging to specifically assess the superior ophthalmic vein was not performed; this is acknowledged as a limitation, since ophthalmic vein enlargement is a recognized indirect marker of orbital venous congestion in dAVF-related proptosis and its status would have added further diagnostic confidence.

**Figure 1:**
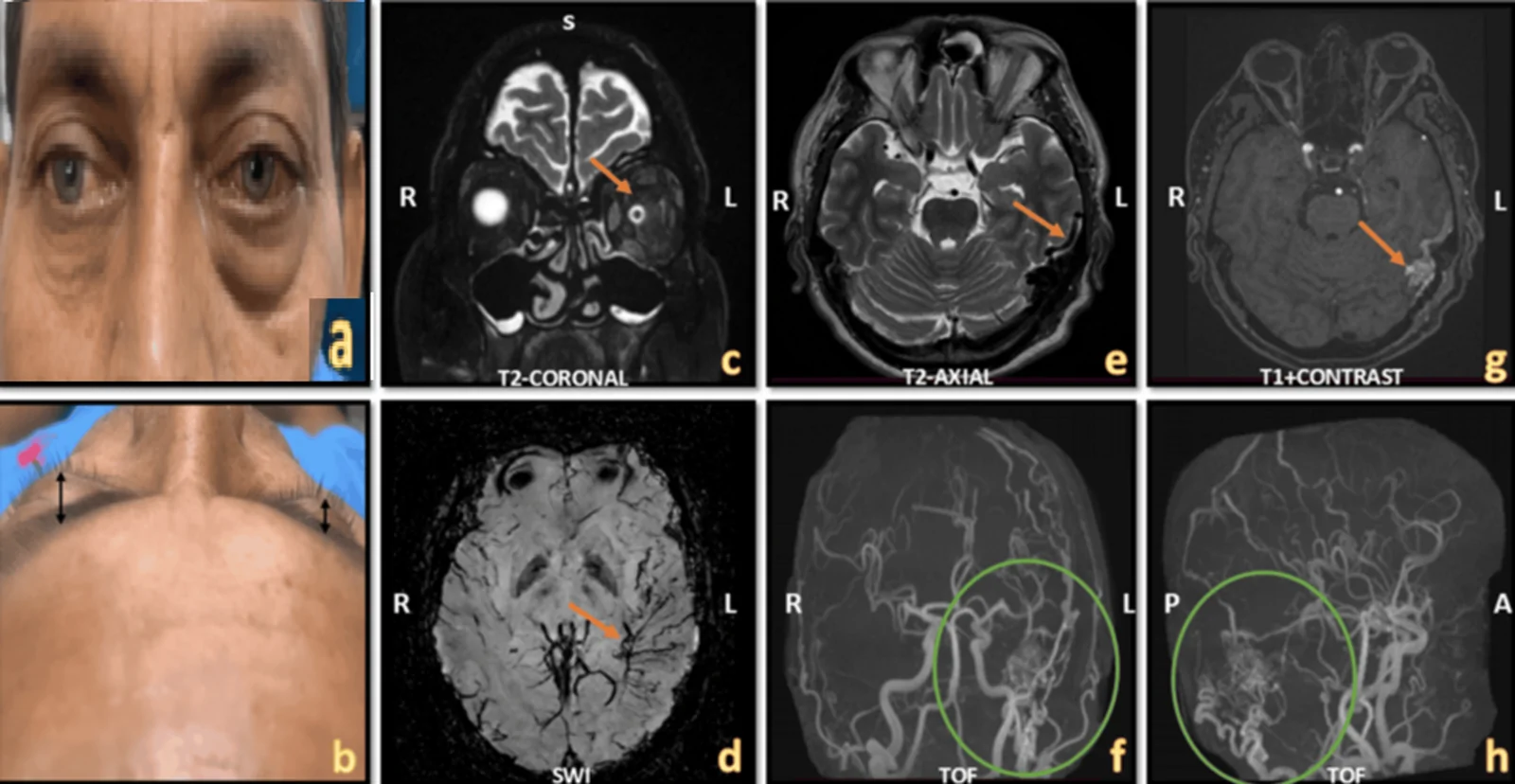
Clinical proptosis and MRI sequences showing left posterior temporal lobe dural arteriovenous fistula (a) Left eye chemosis, swelling, and proptosis. (b) Proptosis visible on superior view. (c) T2-weighted coronal MRI showing swelling in the left orbit. (d) SWI axial section showing increased vascularity on the left compared to the right. (e) TOF-MRA showing a dilated vein of Labbé, visible as a flow void. (f-h) Additional TOF-MRA and T1 post-contrast sequences show multiple dilated flow voids in the left posterior temporal lobe and around the left transverse sinus, with dilated cortical venous channels along the curvature of the left temporal lobe and abnormal enhancement, raising the possibility of communication with the superior division of the left middle cerebral artery SWI: susceptibility-weighted imaging; TOF-MRA: time-of-flight magnetic resonance angiography

On re-examination prompted by these imaging findings, a bruit, not appreciated at initial presentation, was identified on palpation and auscultation of the left occipitotemporal scalp, 4 cm posterior to the left pinna. Local Doppler and transcranial Doppler confirmed mixed arterial and venous flow in this area (Video 1).

**Video 1:** Transcranial Doppler, showing the probe placed at left temporo-occipital area Transcranial Doppler, probe placed at the left temporo-occipital area, demonstrating arterial-type systolic and diastolic pulsatile flow with bidirectional signals and spectral broadening: turbulent, back-and-forth flow consistent with combined arterial and venous flow

Management

The patient was referred to interventional neuroradiology for digital subtraction angiography (DSA) and embolization. He declined further investigation and treatment. As a result, formal Borden/Cognard classification could not be confirmed angiographically; he was counseled on the risks of declining intervention and advised close outpatient follow-up. We obtained written informed consent for publication.

The patient was referred to interventional neuroradiology for DSA and embolization. He declined further investigation and treatment. Given this, formal Borden/Cognard classification could not be confirmed angiographically; he was counseled on the risks of declining intervention and advised close outpatient follow-up.

## Discussion

Isolated unilateral proptosis encompasses a broad differential that must be systematically evaluated through history, examination, and targeted investigation. Among inflammatory etiologies, thyroid-associated orbitopathy, although classically bilateral, presents with apparent unilateral exophthalmos at first evaluation in up to 13.5% of cases [4] and should remain a prime consideration, particularly in middle-aged individuals without other systemic disease. Isolated painful proptosis without other symptoms may suggest idiopathic orbital inflammation (orbital pseudotumor), characterized by extraocular muscle inflammation. Infectious processes, notably orbital cellulitis or fungal involvement such as mucormycosis, present acutely with pain, fever, and motility restriction, frequently following sinusitis. Angiographic confirmation is required for vascular causes of proptosis such as carotid-cavernous fistula and cavernous sinus thrombosis; both of which, along with an orbital mass, were the primary differentials considered and excluded in this patient on the basis of normal cranial nerve findings, normal orbital imaging, and the eventual MRI identification of a temporal dAVF rather than a mass lesion. Neoplastic infiltration, whether primary (e.g., cavernous hemangioma, lymphoma, meningioma, lacrimal gland tumors) or metastatic (e.g., from lung, prostate), should also be considered, especially with insidious onset or mass effect on imaging [5].

A dAVF is an aberrant arteriovenous shunt developing within the dura mater. The clinical picture it produces is dictated by multiple factors, including the degree of arteriovenous shunting, the feeding artery, the fistula's location, and its venous drainage pattern [6]. Clinically, dAVF-related symptoms are broadly classified as hemorrhagic (resulting from CVD and rupture) or nonhemorrhagic (nonhemorrhagic neurological deficit (NHND): focal neurological deficit, seizure, or, as in this case, a localized congestive/mass effect from venous reflux) [7,8].

Carotid-cavernous fistula is the most common type of AVF responsible for ocular signs and symptoms, and it is distinctly unusual for dAVFs in other locations to produce ocular signs. Robert et al. retrospectively studied 13 patients with cranial noncavernous dAVFs presenting with ophthalmic signs and symptoms; 38.5% of these patients had proptosis [9]. Proposed mechanisms by which dAVFs evoke ocular symptoms include local venous reflux to the superior ophthalmic vein, cerebral venous dilatation raising intracranial pressure, venous dilatation compressing the oculomotor nerve, and an intraorbital fistula draining directly into the superior ophthalmic vein [9]. In our case, venous reflux via the vein of Labbé, which is a cortical vein, is the most plausible mechanism for the isolated proptosis, distinct from the superior ophthalmic vein-mediated mechanism more typical of cavernous or anterior fossa dAVFs; direct imaging confirmation of ophthalmic vein involvement was not obtained in this case, as noted above.

Flow pressure in a dAVF can be high or low, depending on insufficient antegrade flow, retrograde flow, drainage into a cortical vein, and the presence of intracranial hypertension [10]. The presence of CVD is the single most important feature placing a dAVF in a higher risk category. In lesions with CVD, an annual mortality rate of 10.4%, an annual risk of intracranial hemorrhage of 8.1%, and an annual risk of NHND of 6.9% have been reported, with a significant difference in annual hemorrhage risk between symptomatic and asymptomatic patients (7.4% vs. 1.5%, respectively) [7,8].

Although DSA is required for definitive Borden/Cognard classification, which was declined by our patient, the pattern seen on MR angiography is informative in its own right. Drainage into both a dural sinus (transverse sinus) and a cortical vein (vein of Labbé) is consistent with Borden type II (equivalently, Cognard type IIa+b or IIb) [1,2], categories in which cortical venous reflux is present and which carry a meaningfully elevated risk of hemorrhage and NHND compared with Borden type I/Cognard type I-IIa lesions. This illustrates that even without angiographic confirmation, noninvasive imaging alone was sufficient to identify this lesion as high risk, which reinforces the urgency of the referral made in this case and should inform counseling when a patient declines DSA.

While DSA remains the gold standard for diagnosing dAVFs, recent advances in CT and MRI have improved noninvasive diagnostic capability by characterizing drainage-vein hemodynamics and identifying adjacent brain injury in suspected dAVF [11]. Though DSA offers near-100% sensitivity and specificity, a meta-analysis of 13 studies reported that MRI (primarily time-resolved MR angiography) achieves a sensitivity of 90% (95% CI: 0.83-0.94) and specificity of 94% (95% CI: 0.90-0.96) for dAVF detection, while CT angiography showed a sensitivity of 80% (95% CI: 0.62-0.90) and specificity of 87% (95% CI: 0.74-0.94) [12]. Anatomic variation in dural venous sinus depressions and drainage patterns can further complicate angiographic interpretation and should be borne in mind when characterizing atypical venous drainage such as that seen here [13].

Treatment of cranial dural AVF is guided by the Cognard classification, based on the direction of dural sinus drainage, the presence or absence of CVD, and venous outflow architecture. There are five types in total: types 1 and 2a are considered benign, while types 2b, 2(a+b), 3, 4, and 5 are considered aggressive with a high risk of rupture. Benign dAVFs are generally managed conservatively with close follow-up, whereas AVFs with high risk of rupture or hemorrhagic conversion require surgical or endovascular treatment, most commonly transarterial or transvenous embolization [14]. Our patient's presumed Borden type II/Cognard type IIa+b-IIb lesion would ordinarily fall into the latter, treatment-indicated category; his decision to decline intervention represents a real-world management challenge and underscores the importance of clear risk communication and close longitudinal follow-up when definitive treatment is deferred.

## Conclusions

Isolated unilateral proptosis can arise from a broad range of etiologies, most of which are more common than dAVF. However, this case illustrates that a temporal dAVF draining via a cortical vein (vein of Labbé), a Borden type II/Cognard type IIa+b-IIb pattern, can present with isolated proptosis in the complete absence of pulsatile tinnitus, cranial neuropathy, or other classical dAVF features. Clinicians should maintain a low threshold for suspecting cranial dural AVF when evaluating isolated proptosis, since apoplectic rupture of high-risk lesions is possible and may necessitate urgent treatment. This case also highlights a real-world limitation: when angiography is declined, noninvasive MR angiographic drainage patterns can still meaningfully inform risk stratification and guide the urgency of counseling. Optimal management of such atypical presentations requires multidisciplinary evaluation involving ophthalmology, neurology, and interventional neuroradiology.
